# “Soft Protein Corona” as the Stabilizer of the Methionine-Coated Silver Nanoparticles in the Physiological Environment: Insights into the Mechanism of the Interaction

**DOI:** 10.3390/ijms23168985

**Published:** 2022-08-11

**Authors:** Aleksandra M. Bondžić, Dunja Jovanović, Nevena Arsenijević, Bojana Laban, Tamara Lazarević Pašti, Urszula Klekotka, Bojan P. Bondžić

**Affiliations:** 1 Vinča Institute of Nuclear Sciences, National Institute of the Republic of Serbia, University of Belgrade, P.O. Box 522, 11000 Belgrade, Serbia; 2 Faculty of Science and Mathematics, University of Priština in Kosovska Mitrovica, Lole Ribara 29, 38220 Kosovska Mitrovica, Serbia; 3 Institute of Chemistry, University of Bialystok, Hurtowa 1, 15-399 Bialystok, Poland; 4 National Institute of the Republic of Serbia, University of Belgrade-Institute of Chemistry, Technology and Metallurgy, Njegoseva 12, 11000 Belgrade, Serbia

**Keywords:** protein corona, AgNPs, BSA, adsorption, kinetics, thermodynamic study

## Abstract

The study of the interactions between nanoparticles (NPs) and proteins has had a pivotal role in facilitating the understanding of biological effects and safe application of NPs after exposure to the physiological environment. Herein, for the first time, the interaction between L-methionine capped silver nanoparticles (AgMet), and bovine serum albumin (BSA) is investigated in order to predict the fate of AgMet after its contact with the most abundant blood transport protein. The detailed insights into the mechanism of interaction were achieved using different physicochemical techniques. The UV/Vis, TEM, and DLS were used for the characterization of the newly formed “entity”, while the kinetic and thermodynamic parameters were utilized to describe the adsorption process. Additionally, the fluorescence quenching and synchronous fluorescence studies enabled the prediction of the binding affinity and gave us insight into the influence of the adsorption on the conformation state of the BSA. According to the best of our knowledge, for the first time, we show that BSA can be used as an external stabilizer agent which is able to induce the peptization of previously agglomerated AgMet. We believe that the obtained results could contribute to further improvement of AgNPs’ performances as well as to the understanding of their in vivo behavior, which could contribute to their potential use in preclinical research studies.

## 1. Introduction

After administration of nanoparticles in the bloodstream, they are exposed to numerous proteins, which mainly determine the NPs’ final physiological response. Proteins’ adsorption on the surface of nanoparticles, called “protein corona”, depends on many factors, such as the pH, size, shape, and charge of the nanoparticles [1]. The influence of nanoparticles’ size on the protein corona formation is probably the most investigated effect, and the general stance is that the NPs with lower diameter adsorb higher amounts of proteins [2,3]. On the other hand, the investigations of the influence of the NPs’ shape pointed out that the shape might influence the conformation state of the adsorbed protein, while the influence on the amount of adsorption was not investigated [4,5]. In addition, the surface charge of NPs plays a crucial role in protein adsorption, and recent data suggest that functionalizing of NPs with negatively charged molecules has certain advantages. While the positively charged NPs are rapidly recognized by opsonins and eliminated from the body, the negatively charged and neutral NPs are retained for a longer time; additional, neutral NPs seem to adsorb a smaller amount of protein than negatively charged NPs [1]. In addition to the NPs’ properties, proteins’ adsorption on the NPs’ surface depends on proteins’ charge and size [6], as well as hydrophobic/hydrophilic character [7].

Regarding the binding strength to the NPs’ surface, the rate of formation, and the stability of the formed NP-protein complex, proteins’ corona can be divided into “hard” and “soft corona” [1,8,9]. The hard corona is characterized by rapid formation of the stable NP-protein complex over time, which is most often able to change the final physiological response of the nanoparticles. On the contrary, the formation of the “soft protein corona” is a slow process, characterized by weak binding of protein on the NPs with decreased ability to influence the NPs’ final physiological properties [10]. Hence, the nature and properties of protein corona and its formation have to be investigated and understood before any translation of new nanomaterials into further biological evaluations and potential clinical trials.

Owing to their fascinating physical and chemical properties, including small-scale effects, optical effects, antimicrobial activity, excellent stability, and biocompatibility, silver nanoparticles (AgNPs) are widely used in biomedical, environmental, and material science [11,12]. After the administration of AgNPs into the bloodstream, they are exposed to transport proteins, primarily serum albumin, forming the so-called “protein corona” entity. As mentioned above, this formed “protein corona” mainly determines the fate of AgNPs under physiological conditions. In the recent publication, Tai et al. investigated the influence of the BSA adsorption on the citrate-stabilized AgNPs with an average monomer diameter of 24 nm in an acidic environment and found that the formed protein corona highly improved colloidal stability by preventing the acid-induced interfacial dissolution of AgNPs and their aggregation by enhanced electrostatic repulsion [13]. In their recently published work, Boehmler et al. investigated size-dependent, BSA-enhanced AgNPs dissolution kinetics in the citrate buffer, pH 6.5 in the presence of up to 2 nM BSA, which provided monolayer formation [14]. They found that the loading of NPs’ surfaces depends on the NPs’ size and that better loading was achieved for larger NPs. However, the dissolution behavior was the most expressed for smaller particles. Kennedy et al. investigated the stabilization of aqueous suspension of citrate silver nanoparticles by human serum albumin (HSA) and compared their toxicity with non-stabilized citrate AgNPs and polyvinylpyrrolidone (PVP) AgNPs [15]. The authors showed that the cyt-AgNPs suspension prepared in buffer solution underwent strong agglomeration and precipitation and thus showed low toxicity compared to the suspension prepared in water and then added to the medium. On the other hand, the toxicity of citrate AgNPs prepared in water was the same as the toxicity of the PVP particles. In the other study, the composition of formed protein corona for the above mentioned types of AgNPs (citrate-stabilized and polyvinylpyrrolidone-stabilized, in two sizes, 20 nm and 110 nm) was examined by utilizing a label-free mass-spectrometry-based proteomics approach [7]. All AgNPs were associated with a common subset of 11 proteins, including albumin, apolipoproteins, keratins, and other serum proteins. They found that bigger AgNPs, both citrate- and PVP-stabilized, bind two times higher the number of proteins compared to the smaller NPs. Such results were explained by differences in protein corona formation based on surface curvature. In addition, the differences were also found in the composition of adsorbed proteins; the smaller NPs adsorbed more hydrophobic proteins, while the protein corona of bigger AgNPs consisted of more hydrophilic proteins.

In our previous study, methionine-coated AgNPs were synthesized for the first time and characterized in detail [16]. It was found that these AgNPs have an average size diameter of about 7.7 nm, and in the aqueous solution, they undergo a mild flocculation process. In order to predict how the most abundant transport protein, serum albumin, could influence this new type of NP, in this study, the effect of BSA adsorption on these small, negatively charged methionine-coated AgNPs was evaluated in the 10 mM phosphate buffer, pH 7.4. BSA was chosen as a model protein because of its similarity to human serum albumin. The protein corona formation and its influence on colloid stability were investigated in detail by performing kinetic, thermodynamic, and adsorption studies. Additionally, the influence of adsorption on the conformation of BSA was investigated using fluorescence and synchronous fluorescence studies. The authors believe that the obtained results could contribute to the further improvement of AgNPs’ performances, to the understanding of the biological effects and safe application of AgNPs in a biological milieu, and also to the understanding of their in vivo behavior contributing to potential preclinical research.

## 2. Results and Discussion

### 2.1. UV/Vis Spectrophotometric Analysis of the Interaction of BSA–L-Met-Coated AgNPs under Physiological-like Conditions

**Figure 1:**
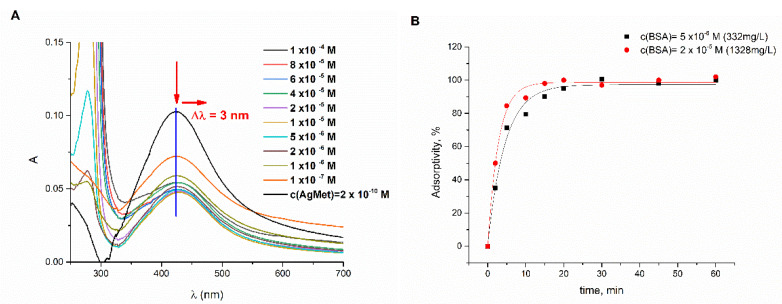
(**A**) The absorption spectra of 2 × 10^−10^ M AgMet in 10 mM phosphate buffer pH 7.4, in the absence (black line) and in the presence of the increasing concentration of BSA (1 × 10^−7^–1 × 10^−4^ M). (**B**) The dependence of the adsorptive capacity of 2 × 10^−10^ M AgNPs as the function of time and the BSA’s concentration.

**Figure 2:**
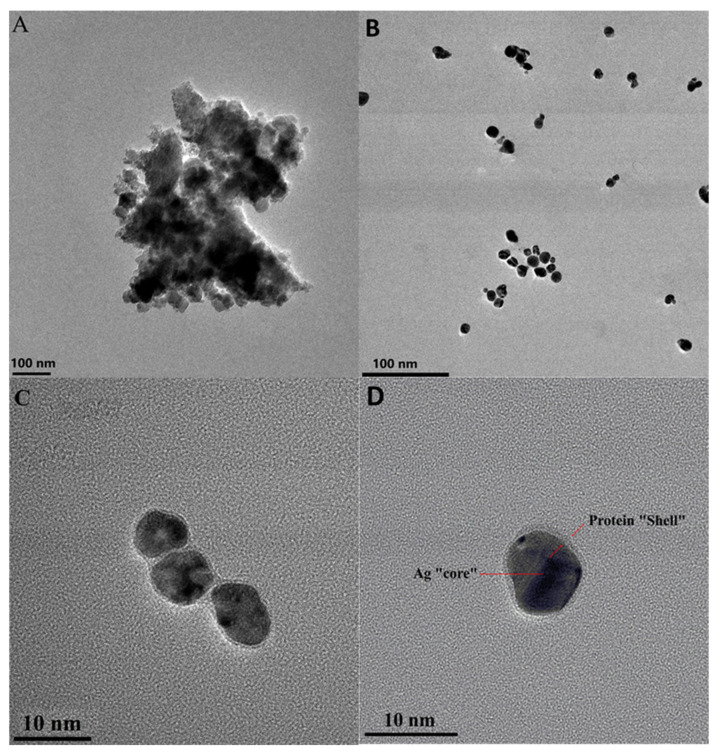
(**A**) TEM micrograph of 2 × 10^−10^ M of methionine-covered AgNPs in the 10 mM phosphate buffer; (**B**) TEM micrograph of observed peptization process after addition of 5 × 10^−6^ M BSA; (**C**) TEM micrograph in the 10 nm resolution; (**D**) silver nanoparticle core and protein shell indicated by arrows.

The interaction between bovine serum albumin and AgMet was investigated using UV/Vis spectroscopy, following the changes in the surface plasmon resonance band (SPR) of these nanoparticles. The solution of 2 × 10^−10^ M methionine-coated AgNPs in 10 mM phosphate buffer was incubated for 3 h with different concentrations of BSA at 37°, and UV/Vis spectra were recorded. The UV/Vis spectrum of AgMet in 10mM phosphate buffer possesses the SPR band with a maximum at about 424 nm and a broad second peak at higher wavelengths associated with the presence of large AgNPs aggregates formed upon addition of phosphate buffer (Figure 1A, black line). However, with the increase of BSA concentration, red (from 424 nm to 427 nm) and hypochromic shifts were observed (Figure 1A), indicating gradual covering of the surface of NPs. The disappearance of the second broad peak at higher wavelengths indicated peptization of self-formed flocs, vide infra (Figure 2B). On the other hand, upon adding 5 × 10^−6^ M BSA, no further spectral changes were observed, implying saturation of AgMet’s surface by BSA. These synchronous changes clearly pointed out the protein binding to the surface of the nanoparticles with corona formation [17].

In order to determine the time necessary for full covering of AgMet surfaces, the equilibrium time, the percentage of absorptivity was evaluated for the different incubation times. The solutions, which contained 2 × 10^−10^ M AgMet in phosphate buffer, pH 7. 4, and 5 × 10^−6^ or 2 × 10^−5^ M BSA, were incubated during 1 h at 37 °C, while the amount of adsorbed BSA was estimated at different time intervals (Figure 1B). Figure 1B shows that the percentage of adsorbed BSA was increasing in the first 30 min. After this time, changes in the adsorbed amount of BSA were not observed, indicating that the equilibrium state was reached. According to Figure 1B, full coverage of AgMet’s surfaces was achieved within 30 min for BSA’s concentration above 5 × 10^−6^ M. An additional increase of BSA’s concentration influenced only the adsorption rate but not the adsorption capacity, which is in accordance with the results obtained from the absorption spectra.

### 2.2. Characterization of Formed BSA/AgMet Conjugates by TEM, Dynamic Light Scattering, and Zeta Potential Measurements

TEM experiments were performed to obtain insights into the size and morphology of prepared AgMet nanoparticles and their conjugates with BSA. In our previously published study, we have shown that these AgNPs have an average diameter of 7.7 nm and undergo a mild flocculation process in an aqueous solution [16]. The self-flocculation in an aqueous solution is probably a consequence of hydrogen bonding between terminal amino groups of the attached methionine on one AgNP and the terminal carboxyl group of methionine attached to the adjacent AgNPs. A similar effect was previously observed between Au nanoparticles modified with lysine, arginine, or cysteine [18]. TEM images of AgNPs dispersed in 10 mM phosphate buffer showed their strong flocculation, disabling the determination of precise sizes of these nanoparticles (Figure 2A). This pronounced flocculation of AgNPs in phosphate buffer could be explained by the sorption of phosphate anions on the surface of AgNPs, which resulted in the increased reduction of the NP surface charge at higher buffer concentrations [19]. Unlike NPs stabilized by amino acids, NPs stabilized by citrate ions do not show a tendency to flocculate in an aqueous solution [15], probably due to their inability to form hydrogen bonds between themselves, while they show similar behavior in the phosphate buffer as AgMet nanoparticles [20]. After the addition of buffered AgMet dispersion in BSA solution, the dispersion underwent the peptization process, which is clearly observed in the TEM micrograph (Figure 2B). Additionally, Figure 2C,D show that AgNPs are surrounded by a non-uniform BSA cloud by forming a core (AgNPs, black sphere)–shell (BSA, white-grey cloud)-type structure [21,22]. Undefined clouds of BSA surrounding the NPs indicate the presence of a “soft” corona [23]. This formed corona has a stabilization effect on the synthesized nanoparticles by disabling reflocculation. Turbay et al. found that use of BSA as a stabilizing agent during synthesis of AgNPs formed a “soft corona” with AgNPs, unlike lysozyme which formed a “hard corona” [6]. These authors showed that “hard protein corona” formation around AgNPs acts as the antagonist of their antibacterial activity, which points out superior properties of the “soft” protein corona. Contrary to Turbay et al., Eby et al. have shown that lysozyme-functionalized AgNPs exhibit potent antimicrobial activity, which could be explained by different methods of synthesis of these AgNPs [24].

As a sensitive tool for particle size measurement, the DLS method has been chosen to confirm protein corona formation after the addition of the BSA in the AgNPs solution. After 30 min of incubation of 2 × 10^−10^ M AgMet with 5 × 10^−6^ M BSA in 10 mM phosphate buffer, the measurements were performed. Zeta potential measurements were also made. As shown in Table 1, the obtained AgNPs’ average hydrodynamic diameter value was 57.08 nm, which, in combination with the high polydispersity index (PDI) (about 0.89), clearly indicated the above-mentioned flocculation process. On the other hand, the BSA’s measured hydrodynamic diameter was 7.93 nm with PDI value 0.1, pointing out the monodisperse system. The significant increase in AgNPs’ diameter observed upon incubation with BSA was consistent with formed conjugates, while the lower index of polydispersity of the formed “protein corona” compared to methionine-stabilized AgNPs confirmed the peptization process. The increase in absolute zeta potential observed upon incubation indicated that the adsorption of negatively charged BSA led to the stabilization of the system. Additionally, the higher absolute mobility and conductivity values indicated that BSA could displace methionine from the hydrodynamic sphere of AgNPs. In our previous study, using density functional theory calculations, it was shown that the methionine is dominantly bound by vertical binding geometry via the terminal amino group, while the horizontal binding mode via sulfide and amino groups is also possible. Probably, there are amino acids in the sequence of BSA, possessing side chains with a higher affinity toward Ag surface than methionine NH_2_- or -S- groups that are able to supersede it from the hydrodynamic sphere of AgNPs. On the other hand, the recently published work pointed out that BSA adsorption enhanced the dissolution of AgNPs through the displacement of Ag(I)(aq)-loaded BSA by excess protein in the bulk solution [14], indicating that this dissolution process could be the reason for the observed significantly increased mobility. In order to verify whether the aforementioned dissolution process contributes to the increase in conductivity of the AgMet/BSA dispersion, the presence of Ag^+^ ions was tested by adding concentrated HCl to the supernatant obtained after corona formation. No turbidity of the solution or precipitation of the formed AgCl was observed after centrifugation, indicating that the increased conductivity was not due to released Ag^+^ ions. This finding indicated that part of the methionine ions was replaced from the hydrodynamic sphere of AgNPs with BSA molecules.

### 2.3. Quenching of Tryptophan Fluorescence of BSA by MET-Coated AgNPs

**Figure 3:**
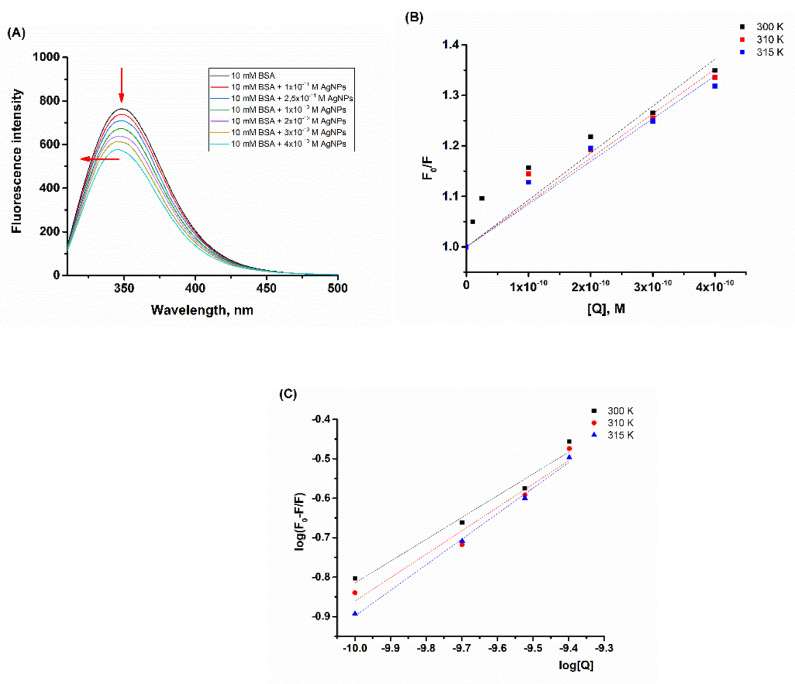
(**A**) The fluorescence spectra of BSA in the absence (black line) and in the presence of the increasing concentration of methionine-coated AgNPs. (**B**) The Stern–Volmer plot for Trp fluorescence quenching induced by the increasing AgMet concentrations obtained at different temperatures (300 K, 310 K, and 315 K). (**C**) Double logarithmic plot of the Trp fluorescence quenching of BSA with different AgMet concentrations. The BSA concentration was 1 × 10^−5^ M in the 10 mM phosphate buffer, pH 7.4. The AgMet concentrations ranged from 1 × 10^−11^ M to 4 × 10^−10^ M. The incubation time was 5 min.

The results presented in the previous chapters indicated that BSA could be adsorbed on the surface of the methionine-coated AgNPs by forming a “soft protein corona” entity. In order to gain deeper insights into the mechanism of interaction, the fluorescence-quenching titration studies as well as the synchronous fluorescence measurements were performed. First, 1 × 10^−5^ M BSA’s solution in 10 mM phosphate buffer pH 7.4, which contained the increasing concentrations of AgNPs, was incubated 5 min before fluorescence measurements. BSA’s fluorescence spectrum in 10 mM phosphate buffer showed one emission maximum at 343 nm. The intrinsic fluorescence of BSA originated from two tryptophan residues. One of them is located on the protein’s surface, Trp-134, while the other, Trp-212, is buried deep in the hydrophobic interior of the protein. After the addition of the investigated AgNPs to the increasing concentrations of up to 2.5 × 10^−11^ M, a decrease in the emission intensity was observed (Figure 3A). This pointed out the intact secondary structure of BSA and indicated that the tertiary structure was affected by AgNPs [25]. At the same time, Trp residues were surrounded by similar environments [26]. In the concentrations above 2.5 × 10^−11^ M AgNPs, with further addition of AgNP, the emission maximum began to change and a blue shift of up to 5 nm followed by a gradual decrease of emission maximum was observed (Figure 3A), signifying the changes in the Trp microenvironment as a result of the structural loss in the protein [25,26].

In order to determine the Stern–Volmer constant of fluorescence quenching, the Stern–Volmer plots (SV plot) were designed by using Equation (1):

(1) $$\frac{F_{0\;}}{F}=1+{\;K}_{\mathrm{sv}}\left[Q\right]=1+k_{q}\tau_{0},$$

where F_0_ and F are the fluorescence intensities in the absence and in the presence of the quencher (AgMet), K_sv_ is the Stern–Volmer constant, [Q] is the concentration of the quencher, AgMet, k_q_ is the bimolecular quenching rate constant, and τ_0_ is the average fluorescence lifetime of the biomolecule (10^−8^ s) [26].

The obtained SV plots exhibited linear behavior (Figure 3B), indicating the possibility of the existence of the static and/or dynamic quenching processes. In order to distinguish the dynamic from the static quenching process, the quenching titration studies were performed at different temperatures. The Stern–Volmer plots obtained at different temperatures differed between themselves in the slopes; with the increasing temperature, the slopes in the Stern–Volmer plots decreased, pointing out the static quenching mechanism (Figure 3B). The fluorescence parameters, the Stern–Volmer, K_sv_, and bimolecular quenching rate, k_q_, constants are shown in Table 2. From Table 2, it can be observed that K_sv_ values decreased with increasing temperature, thus implying that a clearly static process takes place. In addition, the six orders of magnitude higher value for k_q_ compared to k_q_ of the diffusion-controlled process, 1 × 10^10^ M^−1^ s^−1^, was observed, pointing out the existence of some type of binding interaction.

The binding parameters, the binding constant (K), and the number of binding sites (n) were determined graphically (Figure 3C) using Equation (2):

(2) $$\log\left[\frac{F_{0}-F}{F}\right]=\mathrm{logK}+\mathrm{nlog}\left[Q\right],\;$$

where F_0_ and F represent the fluorescence intensities in the absence and in the presence of the quencher, while the thermodynamic parameters have been calculated using van’t Hoff Equations (3) and (4):

(3) lnK=−ΔHoRT+ΔSoR, 

(4) $$\Delta G^{o}=\Delta H^{o}-T\Delta S^{o}$$

where K is the previously calculated binding constant for the corresponding temperature; R is the molar gas constant. The obtained values for ΔH°, ΔS°, and ΔG° are shown in Table 2. The binding constant K pointed out moderate binding. The negative sign for the Gibbs free energy, ΔG°, indicated the spontaneous binding process between BSA and AgNPs, which was much more favorable at higher temperatures. The positive sign obtained for ΔH° and ΔS° pointed to the entropy-driven and endothermic process induced by hydrophobic forces [27]. Hydrophobic interactions during adsorption of BSA on AgNPs were previously reported by Ravindran et al. [28], Mariam et al. [27], Waghmare et al. [29], Wang et al. [17], etc. On the other hand, several publications point to electrostatic interactions as the main forces between BSA and AgNPs [6,30,31]. Jiang et al. investigated interactions between BSA and citrate-stabilized AgNPs in aqueous solution using fluorescence spectroscopy [30]. They observed blue shift of emission maximum which they associated with potential aggregation of protein upon addition of AgNPs, but no evidence was presented to confirm this statement. On the contrary, in this work we found that the hydrophobic forces were responsible for interaction as well as protein corona formation (Figure 2) instead of protein aggregation, which is in good agreement with the previously published paper [29].

**Figure 4:**
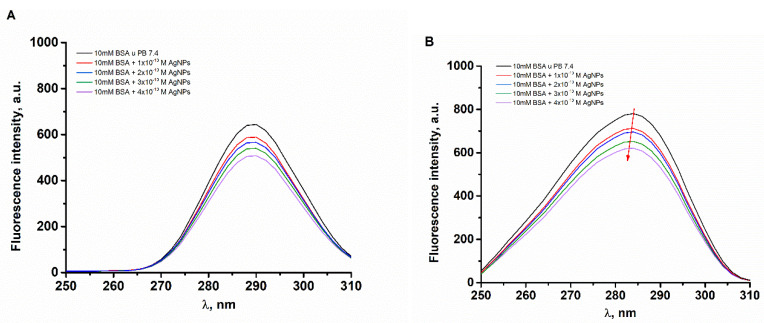
The synchronous fluorescence spectra of 1 × 10^−5^ M BSA in the absence and the presence of increasing AgMet concentration (1 × 10^−10^–4 × 10^−10^ M) in 10 mM phosphate buffer, pH 7.4. (**A**) Δλ = 15 nm and (**B**) Δλ = 60 nm.

The synchronous fluorescence study was performed in order to estimate the effect of the binding of AgMet nanoparticles on the conformational changes of the BSA. In Figure 4, the synchronous fluorescence spectra of 1 × 10^−5^ M BSA at Δλ = 15 nm and Δλ = 60 nm are shown, respectively. When Δλ = 15 nm (Figure 4A), there are no changes in the position of emission maximum, indicating that the microenvironment of tyrosine residues stayed intact. On the other hand, it is clear that the emission peaks of Trp’s residues are slightly blue-shifted, indicating that AgNPs induced such BSA conformation changes and that Trp residues are now buried deeper in the hydrophobic environment of protein [32]. This finding is in good agreement with most published works.

### 2.4. Adsorption Isotherm and Kinetic Analysis

**Figure 5:**
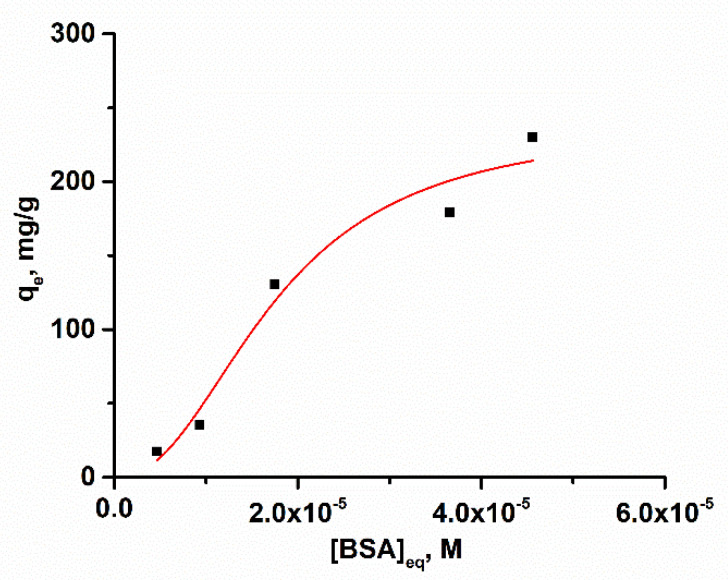
The adsorption isotherm for BSA adsorption on the surface of methionine-coated Ag nanoparticles.

In order to obtain additional information about the interaction, an adsorption isotherm was made. The different concentrations of BSA, 5 × 10^−6^–50 × 10^−6^ M were incubated with 2 × 10^−10^ M AgMet at 37° during 30 min, and the amount of unabsorbed BSA retained in the supernatant was determined spectrophotometrically after centrifugation. The dependence of the concentration retained in the adsorbent phase, q_e_, in the function of BSA equilibrium concentration is presented in Figure 5.

For describing the adsorption process of BSA on the surface of investigated AgNPs, Hill isotherm, presented by Equation (5) was used [33]:

(5) $$q_{e}=\frac{q_{\mathrm{SH}}{\left[\mathrm{BSA}\right]}_{e}^{n}}{K_{D}+{\left[\mathrm{BSA}\right]}_{e}^{n}}$$

where q_e_ is the equilibrium adsorption capacity, q_SH_ is Hill isotherm uptake capacity, K_D_ is Hill isotherm constant, and n is Hill cooperativity coefficient of the binding interaction. Based on the shape of the adsorption isotherm, multilayer adsorption is evident, with Hill maximum uptake capacity, q_SH_ = (239 ± 55) mg/g and n = 2.25 ± 1.10. Multilayer adsorption associated with moderate isotherm constant K_D_ (1.76 ± 0.57) × 10^−5^ M^−1^ confirmed “soft corona” formation. Multilayer adsorption of BSA is also observed by Waghmare et al. [29].

The kinetics of adsorption of BSA onto the surface of AgMet was elucidated by the pseudo-first-order (Equation (6)), the pseudo-second-order (Equation (7)), and the intraparticle diffusion models (Equation (8)), while R^2^ (the correlation coefficients) values were used to evaluate which model was better for describing observed adsorption kinetics.

The pseudo-first-order, the pseudo-second-order, and the intraparticle diffusion models are expressed by Equations (6)–(8), respectively:

(6) $$\log\left(q_{e}-q_{t}\right)={\mathrm{logq}}_{e}-k_{1}t\;$$

(7) $$\frac{t}{q_{t}}=\frac{1}{k_{2}q_{e}^{2}}+\frac{t}{q_{e}}$$

(8) qt= kit12+C

where q_e_ and q_t_ are the amount of BSA (mg/g) adsorbed on the AgMet surface at equilibrium and at time t; k_1_, k_2_, and k_i_ are rate constants of pseudo-first-order, the pseudo-second-order, and intraparticle diffusion rate constant, respectively.

Compared with the pseudo-first-order model, the pseudo-second-order kinetic model fitted better with the experimental data, with the correlation coefficients higher than 0.9999 (Table 3). Additionally, the experimentally obtained q_e_ value was more consistent with q_e_ value calculated using the pseudo-second-order kinetic model than those obtained from the pseudo-first-order model. These results suggest that BSA adsorption on AgNPs follows the pseudo-second-order kinetic model. To further understand the adsorption kinetics, the intraparticle diffusion kinetics model was used to elucidate the diffusion mechanism and to obtain the intraparticle diffusion rate constant k_i_. The obtained plot passed through the origin, indicating that intraparticle diffusion is the sole rate-controlling step [34].

## 3. Materials and Methods

Bovine serum albumin, L-methionine, and silver nitrate were purchased from Merck. Diammonium phosphate and ammonium dihydrogen phosphate were obtained from J. T. Baker Chemical Company. First, 2 × 10^−4^ M BSA stock solution was prepared by dissolving 256.5 mg of the protein in 20 mL phosphate buffer pH 7.4 (10 mM). The stock solution was stored in a refrigerator at 4 °C and was used to prepare a lower concentration of BSA solution. The pH value of the solution was measured with a pH meter Consort C830.

### 3.1. Synthesis of Silver Nanoparticles

The methionine-coated silver nanoparticles (AgMet) were synthesized according to our previously published procedure [16]. Namely, for the reduction of Ag^+^ ions from an aqueous solution of the silver nitrate, L-methionine was used. At the same time, L-methionine was the stabilizing agent. The concentration of L-methionine-coated silver nanoparticles AgMet in stock dispersion was approximately determined using Equation (9):

(9) $$C_{\mathrm{NPs}}=\frac{C_{M}M_{\mathrm{Ag}}}{\frac{4}{\;3}{\;r}^{3}{\pi \rho \;N}_{0}}$$

where $C_{M}$ is the AgNO_3_ molar concentration, M_Ag_ is the Ag molar mass, r—an average particle size radius, ρ—the Ag density (10.49 g cm^−3^), and N_0_—the Avogadro number. For further work, diluted solutions in the 10 mM phosphate buffer, pH 7.4 were used. Final concentration was 1.06 × 10^−9^ M.

### 3.2. Batch Mode Adsorption Studies

The effect of incubation time on the adsorption of BSA on the surface of AgMet (1.06 × 10^−10^ M) was studied in a batch mode of operation during 3, 5, 10, 15, 20, 30, 45, and 60 min at 37 °C in 10 mM phosphate buffer pH 7.4 by gently shaking at 150 rpm. The effect was examined for two different concentrations of BSA (5 × 10^−6^ M and 2 × 10^−5^ M). After incubation, the solutions were centrifuged for 10 min at 14,500 rpm and the obtained supernatant was carefully separated from the pellets. The concentration of BSA retained in the supernatant represented the unadsorbed BSA. Its concentration was determined spectrophotometrically using the molar extinction coefficient for BSA at 280 nm, ε_280_ = 43,800 M^−1^ cm^−1^ [35]. The amount of the adsorbed BSA was calculated from the difference between the initial and non-adsorbed BSA concentrations. The adsorption percentage was calculated using Equation (10):

(10) $$\mathrm{Adsorptivity}\;\left(\%\right)=\frac{C_{i}-{\;C}_{f}}{C_{i}}\times 100$$

where C_i_ and C_f_ represent the initial and final BSA concentrations after the incubation time, respectively. The retained BSA concentration in the adsorbent phase (q_e_, mg/g) was calculated by Equation (11):

(11) $$q_{t}=\frac{\left(C_{i}-C_{t}\right)V}{W}$$

where C_i_ and C_f_ are the initial and BSA concentration at time t (mg/L), V is the volume of solution (L), and W is the mass of adsorbent (g). Each experiment was performed in triplicate.

### 3.3. UV/Vis Measurements

The absorption spectra were recorded on UV/Vis spectrophotometer (Lambda 35, Perkin Elmer) in the wavelength range from 250 to 700 nm.

### 3.4. Transmission Electron Microscopy

Transmission electron microscopy (TEM) was used to determine the morphology and size distribution of nanoparticles. Solution of 5 × 10^−6^ M BSA and 2 × 10^−10^ AgMet in 10 mM phosphate buffer was incubated during 30 min at 37 °C by gentle shaking (160 rpm). After the incubation period, the drop of the suspension was placed onto the surface of a 400-mesh Cu grid coated with a thin layer of amorphous carbon film and left to dry on the air. Afterward, the specimen was stained with 10 μL of 1.5% (*w*/*v*) phosphotungstic acid solution (pH = 7.4) for 2 min, and then the staining solution was drawn away from the edge of the grid with filter paper. The grid was washed with 10 μL of deionized water three times and dried at room temperature. TEM measurements were performed on Tecnai^TM^ type G2 X-TWIN type from FEI (Hillsboro, OR, USA) operated at voltage of 200 kV.

### 3.5. Dynamic Light Scattering and Zeta Potential Measurements

Particle size distribution, zeta potential (ζ), conductivity, and mobility were measured on a Nano-ZS Zetasizer with a 633 nm He–Ne laser (Malvern Instruments, Malvern, UK) and data were analyzed by Zetasizer Software Version 6.20 (Malvern Instruments, Malvern, UK). Before the measurements, all solutions were incubated for 30 min at 37 °C by shaking at 150 rpm, and were filtered through 0.2 μm syringe filter.

### 3.6. Fluorescence Measurements

The fluorescence measurements were performed on Agilent Cary Eclipse fluorescence spectrophotometer. Fluorescence-quenching spectra were recorded in the wavelength range 320–450 nm upon excitation at 295 nm at 300, 310, and 315 K. Excitation and emission slit widths were set at 5 nm, scanning rate was set at 9600 nm/min. For synchronous fluorescence scans, the wavelength range was set from 250 to 500 nm, while the wavelength shift Δλ was 15 nm (for tyrosine residues) and 60 nm (for tryptophan residues). Emission slit widths were set at 10 nm for both tyrosine and tryptophan residues, but the excitation slit was set at 5 nm for tyrosine and 2.5 nm for tryptophan residues.

## 4. Conclusions

In conclusion, this work has shown that under physiological-like conditions, adsorption of BSA molecules on the L-Met-coated AgNPs leads to system stabilization, which is reflected in the breaking of AgNPs’ flocks. Detailed insights into the mechanism of interaction predicted the “soft protein corona” formation. The adsorption is multilayer and is dependent on the contact time and initial protein concentration. Adsorption kinetics follows the pseudo-second-order kinetics, and intraparticle diffusion is the rate-controlling step. The methionine-coated AgNPs have a strong ability to quench the intrinsic tryptophan fluorescence of bovine serum albumin. The fluorescence quenching is the consequence of the static mechanism of quenching. Adsorption is followed by BSA’s conformational changes, which lead to the deep burring of Trp residues in the hydrophobic environment of protein. The binding constants point out weak to moderately strong interactions, while the thermodynamic parameters indicate the spontaneous process where hydrophobic interactions play a pivotal role. The “soft protein corona” is probably a consequence of the small size of AgNPs and the lower affinity of the BSA toward the negatively charged surface of AgNPs at pH value higher than its isoelectric point. However, in addition to that, the formation of the soft corona leads to stabilization of the system; it could enable unchanging bioidentity of investigated AgNPs and facilitate the interaction of “bare” AgNPs with the target molecules. Additionally, while in the previously published papers, BSA was utilized as a stabilizing agent during the synthesis of AgNPs or was added in the aqueous solution in which NPs were not aggregated, in this work, according to the best of our knowledge, for the first time, we have shown that BSA can be used as an external stabilizer agent able to induce the peptization of previously agglomerated AgMet in the physiological environment. Hence, the obtained results could contribute to further improvement of AgNPs’ performances, to the understanding of their biological effects and safe application in a biological milieu, and to the understanding of their in vivo behavior, contributing to potential preclinical research.

## Figures and Tables

**Table 1:** The hydrodynamic diameter (d), zeta potential (ζ), mobility, and conductivity obtained by DLS and zeta potential measurements. In the final concentration, the solution contained 2 × 10^−10^ M AgMet and 5 × 10^−6^ M BSA in 10mM phosphate buffer, pH 7.4.

	d_sr_, nm	ζ, mV	Mobility, μm cm/Vs	Conductivity, mS/cm	PDI Index
BSA	7.93 ± 0.09	−20.7 ± 1.3	−1.62 ± 0.06	1.180 ± 0.095	0.10
AgNPs	57.08 ± 0.15	−25.8 ± 0.8	−2.03 ± 0.07	0.068 ± 0.001	0.89
AgMet/BSA	68.44 ± 0.20	−31.1 ± 2.7	−2.44 ± 0.21	2.580 ± 0.168	0.25

**Table 2:** The Stern–Volmer quenching constants, K_sv_, bimolecular quenching rate constants, k_q_, and the binding parameters for the interaction of AgNPs with BSA at the different temperatures.

Temperature (K)	300	310	315
**K_SV_ (M)**	(9.28 ± 0.61) × 10^8^	(8.79 ± 0.81) × 10^8^	(8.46 ± 0.62) × 10^8^
**k_q_ (M^−1^ s^−1^)**	(8.7 ± 0.6) × 10^16^	(8.9 ± 0.8) × 10^16^	(9.3 ± 0.6) × 10^16^
**K (M^−1^)**	(5.13 ± 0.20) × 10^4^	(1.17 ± 0.10) × 10^5^	(3.80 ± 0.21) × 10^5^
**n**	0.55 ± 0.06	0.59 ± 0.08	0.65 ± 0.03
**ΔH° (kJ mol^−1^)**		98.64	
**ΔS° (JK^−1^ mol^−1^)**		418.11	
**ΔG° (kJ mol^−1^)**	−26.75	−30.93	−33.03

**Table 3:** Kinetic parameters of pseudo-first-order and pseudo-second-order models and the Weber–Morris intraparticle diffusion model for BSA on methionine-stabilized AgNPs ([BSA]_0_ = 1 × 10^−5^ M, [AgNPs] = 1.27 mg L^−1^, T = 310 K, pH 7.4).

Pseudo-first-order model	k_1,_ min^−1^	0.0734 ± 0.0094
	q_e_, mgg^−1^	1023 ± 25
	R^2^	0.2598
Pseudo-second-order model	k_2,_ min^−1^	(1.95 ± 0.01) × 10^−4^
	q_e_, mgg^−1^	250 ± 4
	R^2^	0.9999
Intraparticle diffusion model	k_I,_ min^−1^	0.0019 ± 0.0006
	I, mgg^−1^	303 ± 19
	R^2^	0.9496
